# Catechol/m-Phenylenediamine Modified Sol–Gel Coating with Enhanced Long-Lasting Anticorrosion Performance on 3003 Al Alloy

**DOI:** 10.3390/molecules29194644

**Published:** 2024-09-30

**Authors:** Keqi Huang, Xin Huang, Liyan Wang, Sifan Tu, Zi Yang, Honglei Guo, Bing Lei, Zhiyuan Feng, Guozhe Meng

**Affiliations:** 1 School of Chemical Engineering and Technology, Sun Yat-sen University, Zhuhai 519082, China; huangkq8@mail2.sysu.edu.cn (K.H.);; 2 Department of Materials Science and Engineering, The Ohio State University, Columbus, OH 43210, USA

**Keywords:** corrosion, sol–gel, coating, Al Alloy

## Abstract

Aluminum alloys, characterized by their low density and high mechanical strength, are widely applied in the manufacturing sector. However, the application of aluminum alloys in extreme environments presents severe corrosion challenges. Sol–gel organic coating techniques have garnered significant attention due to their excellent stability, barrier properties, and cost-effectiveness, as well as their simpler processing. Nevertheless, conventional sol–gel coatings are unable to withstand the corrosive effects of high-chloride and high-halide ion environments such as marine conditions, owing to their inherent structural defects. Therefore, this study proposes the utilization of a simple method to synthesize catechol (CA) and meta-phenylenediamine (MPD)-derived catecholamine compounds to modify sol–gel coatings. Surface characteristics of the modified coatings were analyzed using Fourier-transform infrared spectroscopy (FT-IR), ultraviolet-visible (UV-Vis) spectroscopy, scanning electron microscopy (SEM), and X-ray photoelectron spectroscopy (XPS). The thickness of the modified coating was approximately 6.8 μm. The CA/MPD-modified substance effectively densifies the sol–gel coating, enhancing its corrosion protection performance. A 3.5 wt% NaCl solution was used to simulate a marine environment, and electrochemical impedance spectroscopy (EIS) was conducted using an electrochemical workstation to evaluate the coating’s protective properties over a long-term period. The results indicate that the modified coating provides protection for 3003 aluminum alloy for a minimum of 30 days under corrosive conditions, outperforming unmodified sol–gel coatings in terms of corrosion resistance.

## 1. Introduction

Aluminum is a lightweight metal with a density of approximately 2.7 g/cm^3^, about one-third that of steel, making it ideal for producing lightweight products [1,2]. Aluminum alloys play an indispensable role in various industrial sectors, including machinery, electronics, transportation, aerospace, marine engineering, composite materials, and automation materials [3,4]. They are particularly crucial in key components where lightweight properties are highly demanded, such as in aviation, automobile manufacturing, and shipbuilding. Additionally, due to increasing environmental regulations, green development demands, and ongoing advancements in industrial technology, the demand for high-efficiency, energy-saving aluminum alloys is rising, and their applications are expanding significantly.

Although aluminum alloys can form a dense oxide film in the air, they still face severe corrosion problems in industrial applications, especially under extreme conditions [5]. In complex and variable marine environments, characterized by fluctuating temperatures, humidity, and salt spray concentrations, aluminum alloys can undergo various types of corrosion in different regions [6]. Marine vessels, offshore engineering platforms, construction equipment, harbors, docks, and cross-sea bridges made of aluminum alloys are continuously exposed to marine environments, either submerged in seawater or exposed to the marine atmosphere, which makes them particularly susceptible to corrosion failure [7,8]. Consequently, the structural integrity of aluminum alloy materials is prone to wear and damage, leading to a significant reduction in service life and severe deterioration of their normal performance. Prolonged exposure to these corrosive factors can result in resource wastage, increased maintenance costs, and potentially serious safety risks, causing substantial economic losses and accidents. Thus, improving the corrosion resistance of aluminum alloys and extending their service life under harsh marine conditions is a critical challenge that urgently needs to be addressed.

To address the corrosion protection of aluminum alloys, several commonly used methods include cathodic protection, anodizing, corrosion inhibitors, micro-arc oxidation, electroplating, chemical conversion coatings, and organic coatings [9,10,11,12,13,14,15,16]. Among these, organic coatings offer superior corrosion resistance compared to conventional metal coatings and are characterized by excellent shielding properties, stability, ion recognition, low cost, effectiveness, and relatively simple processes [17,18]. Organic coatings are widely applied in complex marine atmospheric environments and are considered one of the best corrosion protection measures. Among various organic coatings, sol–gel coatings stand out for their environmental friendliness, thinness, and good durability, showing great potential for application [19,20,21].

The synthesis of sol–gel coatings typically involves using metal alkoxides as precursors, or specific salts, hydroxides, and complexes [22]. Taking tetraethyl orthosilicate (TEOS) as an example, the main reaction steps in the sol–gel process involve dissolving TEOS in a solvent (usually deionized water or ethanol). Under appropriate conditions (acidic or alkaline), the solute undergoes hydrolysis or alcoholysis to form silanol, resulting in a homogeneous solution. The hydrolyzed silanol then undergoes a polycondensation reaction, forming a network structure of siloxane bonds (Si-O-Si). During this process, the silanol molecules interconnect to form long-chain or three-dimensional polymer structures, known as sol. Under suitable conditions (such as adjusting pH, adding catalysts), the siloxane bonds in the sol undergo further cross-linking to form a more stable gel structure. During gelation, the sol gradually becomes viscous and solidifies into a gel system. The sol system transitions into a gel system through evaporation and drying processes. The gel can then be uniformly applied to the surface of aluminum alloys using methods such as dipping, spraying, or brushing. The silanol groups in the sol (-Si-OH) react with hydroxyl groups on the aluminum alloy surface (-OH) under hydrogen bonding (Si-H) to form Si-O-Al bonds [23]. This bond formation creates a layer of silicon oxide on the aluminum alloy surface, which helps improve coating adhesion and enhance the binding strength between the coating and the substrate, ultimately improving corrosion resistance. However, sol–gel coatings generally have poor long-term corrosion resistance due to their inherent properties. The coating preparation process involves dehydration and polycondensation, which inevitably leads to defects in the coating. Therefore, densifying and modifying the coating is crucial for enhancing the overall performance of sol–gel coatings.

**Figure 1:**
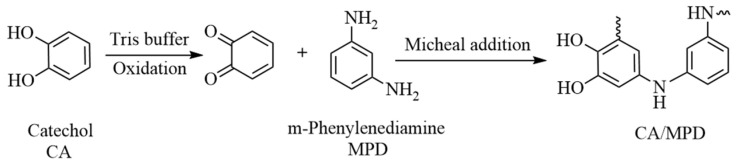
The reaction path of CA and MPD.

To extend the corrosion resistance and service life of sol–gel coatings, modification methods must be employed. For example, Santana et al. [24] prepared SiO₂ sol coatings modified with cerium nitrate and clay particles on low-carbon steel, demonstrating that both modifications could improve the corrosion resistance of the substrate sol–gel coating. Hernández-Barrios et al. [25] found that adding an appropriate amount of cerium nitrate to TEOS-GPTMS sol–gel coatings resulted in products with enhanced barrier properties, suppressing the corrosion activity of damaged areas of the coating and forming a continuous, uniform coating that significantly improved corrosion resistance. Li et al. used mussel-inspired catecholamine substances with strong adhesion properties to modify sol–gel coatings, where the catecholamine material filled the micropores or nanopores in the SiO_2_ sol–gel system, effectively inhibiting the diffusion of corrosive media [26]. They prepared a series of modified sol–gel coatings applied to AZ31 magnesium alloys. Recently, Qiu et al. discovered that catechol/m-phenylenediamine (CA/MPD) could achieve coprecipitation through the Michael addition reaction [27]. The combination of CA/MPD can form a uniform and dense coating on the surface of the substrate, and this coating can effectively fill the nano-defects of the sol–gel structure to increase the barrier effect. The reaction paths of CA and MPD are shown in Figure 1. This nanoscale polymerization process could achieve densification of the substrate, potentially applicable to improving sol–gel system defects and enhancing the corrosion resistance of coatings.

Inspired by these studies, this research designed and prepared a new environmentally friendly catecholamine-modified sol–gel coating to extend the corrosion protection effect and duration on 3003 aluminum alloy. Catechol (CA) and m-phenylenediamine (MPD) were used to synthesize catecholamine substances to modify the sol–gel system. The corrosion protection effect of the modified coating in a 3.5 wt% NaCl aqueous solution was evaluated using electrochemical impedance spectroscopy (EIS) and potentiometric polarization tests. The surface characteristics of the modified coating were analyzed using ultraviolet-visible spectrophotometry (UV-Vis), Fourier transform infrared absorption spectroscopy (FT-IR), scanning electron microscopy (SEM), and X-ray photoelectron spectroscopy (XPS) to elucidate the film formation mechanism and corrosion protection mechanism of the catecholamine-modified sol–gel coating.

## 2. Results and Discussion

### 2.1. Long-Term Corrosion Protection of CA/MPD@sol–gel on Aluminum Alloy

Electrochemical impedance spectroscopy (EIS) is an important method for studying the electrochemical behavior at material interfaces, and it can be used to evaluate the corrosion protection performance of coatings. The impedance at low frequencies (|Z|_f = 0.01Hz_) effectively reflects the corrosion resistance of the coating, where a higher value generally indicates better corrosion protection, and a lower value suggests poorer corrosion resistance. In this experiment, sol–gel samples were immersed in three different concentrations of CA/MPD solutions (0.1 mg/mL, 2 mg/mL, 4 mg/mL) for 14 h for modification, and long-term EIS testing was conducted on sol–gel samples and the three modified samples in a 3.5 wt% NaCl solution environment. In this study, the |Z|_f = 0.01Hz_ value of a blank 3003 aluminum alloy substrate immersed in 3.5 wt% NaCl solution for 5 min (39.4 kΩ) was defined as the critical value for coating failure. When the low-frequency impedance falls below this value, it is considered to have completely lost its protective ability, indicating coating failure. The day the coating is applied is defined as day 0 (0d), the next day as 1d, and so on. The results of the long-term EIS tests for the four groups of samples are analyzed below.

**Figure 2:**
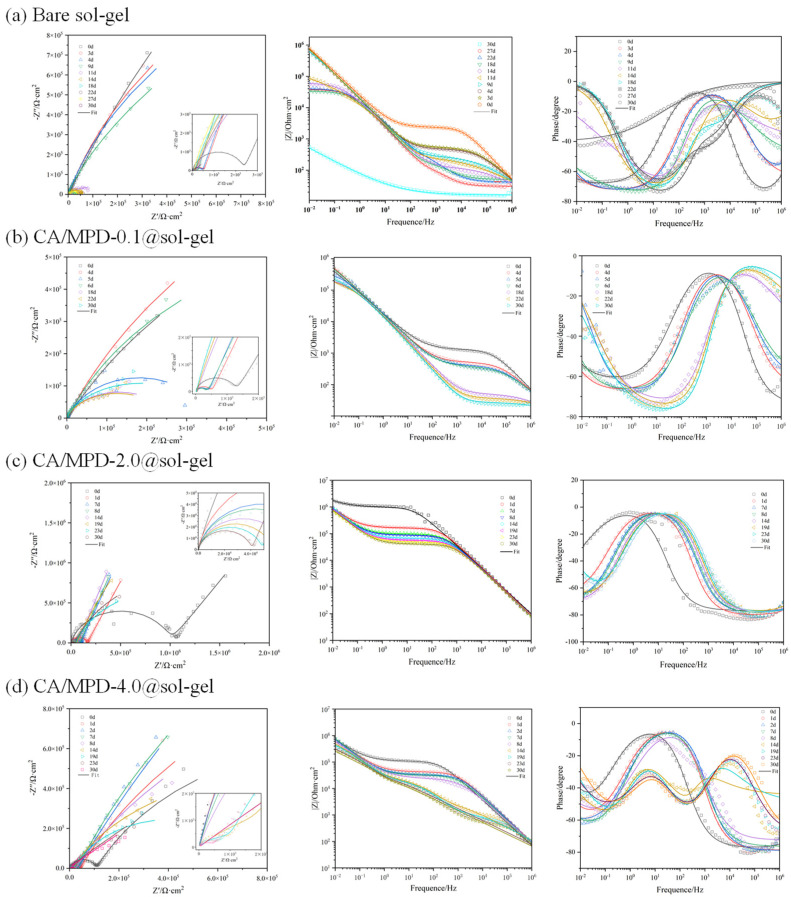
EIS results from different sol–gel coatings on the surface of the aluminum alloy. (**a**) Bare sol–gel, (**b**) CA/MPD-0.1@sol–gel, (**c**) CA/MPD-2.0@sol–gel, (**d**) CA/MPD-4.0@solgel. The results showed that the best anticorrosion performance of the coating was achieved when the concentration of CA and MPD was 2.0 mg/L.

As shown in Figure 2a, the unmodified group demonstrates that the |Z|_f = 0.01Hz_ value of the sol–gel coating decreases continuously under immersion in 3.5 wt% NaCl solution, reaching below the value of the blank substrate by the 30th day, indicating coating failure. In Figure 2b, the CA/MPD-0.1@sol–gel coating provides at least 30 days of protection without showing signs of coating failure. This result suggests that the modification of sol–gel coatings with CA/MPD is feasible and can provide at least 30 days of protection for aluminum alloy materials. As shown in Figure 2c, the EIS results of the CA/MPD-2@sol–gel show that both Bode and Nyquist plots remain stable, with an initial value (0d) of 1768 kΩ, approximately 45 times that of the aluminum alloy substrate. The long-term test shows stability in the range of 900–1000 kΩ, suggesting that this coating can provide corrosion protection for more than 30 days and potentially up to 50 days. Consequently, it can be inferred that the 2 mg/mL CA/MPD-modified sol–gel coating significantly enhances overall corrosion protection performance and increases coating stability. Compared to the CA/MPD-0.1@sol–gel sample, the coating exhibits noticeably improved protective performance and stability. When the concentration of the CA/MPD modification solution is increased to 4 mg/mL, as shown in Figure 2d, its protective performance does not improve compared to the CA/MPD-2@sol–gel group; instead, the corrosion protection performance slightly decreases. A possible reason is that excess CA-MPD diffuses into the microporous structure of the sol–gel, increasing internal stress and reducing the protective effect. Therefore, within a certain range, moderately increasing the concentration of the CA/MPD modification solution can enhance coating stability and improve corrosion resistance, but excessive concentrations may weaken the coating’s corrosion protection capability.

**Figure 3:**
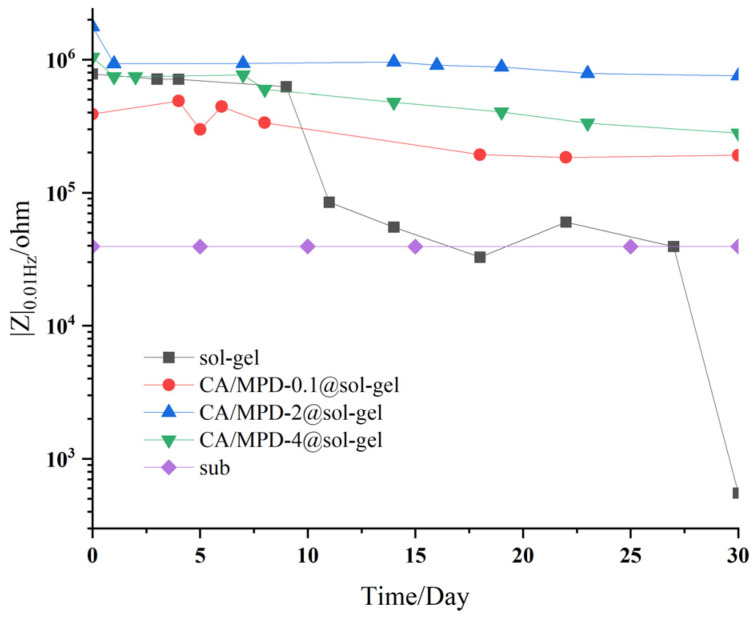
Variation of low-frequency impedance (|Z|_f = 0.01Hz_) over time for sol–gel and different CA/MPD-modified sol–gel coatings immersed in a 3.5 wt% NaCl solution. It can be seen that the values of |Z|_0.01Hz_ decrease slowly with the increase of soaking time, and CA/MPD-2.0@sol–gel has the best anticorrosion performance.

Figure 3 shows the change in low-frequency impedance (|Z|_f = 0.01Hz_) over time obtained from long-term EIS tests for sol–gel samples and three concentrations of CA/MPD-modified sol–gel samples. As the |Z|_f = 0.01Hz_ value can reflect the level of corrosion protection performance, it typically decreases gradually during long-term immersion in a 3.5 wt% NaCl solution for EIS testing [28,29]. The 30-day test results show that the corrosion protection performance of CA/MPD-modified sol–gel coatings remains relatively stable, with the values declining slowly over time. In contrast, the values for sol–gel samples decrease significantly, and corrosion protection performance deteriorates rapidly, dropping to near the blank substrate by the 22nd day, with evident coating failure by the 30th day. This could be due to localized damage or the microporous structure of the sol–gel coating, allowing NaCl solution to penetrate the coating and reach the aluminum alloy substrate, causing corrosion reactions.

For the CA/MPD-0.1@sol–gel sample, the initial |Z|_f = 0.01Hz_ value is lower than that of the sol–gel sample, but its performance retention is better than the sol–gel sample, and no coating failure occurs during the 30-day test. This phenomenon indicates that although the initial protective effect of the CA/MPD-0.1@sol–gel sample is inferior to that of the sol–gel sample, its coating stability is higher under prolonged corrosive environmental immersion, leading to better performance retention. This may be due to the impact of immersion on the sol–gel coating thickness during modification. The initial corrosion protection of the sol–gel coating is significantly influenced by its coating thickness, resulting in lower initial protection performance for the CA/MPD-0.1@sol–gel sample. However, CA/MPD effectively fills the micropores or nanopores in the coating, enhancing its compactness and stability.

Among the three CA/MPD-modified samples, the CA/MPD-2@sol–gel sample shows the best corrosion protection performance. Its value does not decrease significantly over 30 days, while a slow decline is observed in the other two groups. The CA/MPD-4@sol–gel sample exhibits the second-best corrosion protection performance, possibly because the high concentration CA/MPD solution significantly affects the sol–gel coating thickness. Excessive catecholamine material diffusion into the microporous structure increases internal stress, creating new defects, and although the modification improves protection performance and stability, the combined effects result in lower performance than the 2 mg/mL group. The CA/MPD-0.1@sol–gel sample exhibits the lowest corrosion protection performance, likely because the effect of low concentration CA/MPD immersion on the coating thickness is greater than its impact on corrosion resistance, resulting in lower initial protection performance. However, due to the modification effect of catecholamine material, the stability of the sol–gel coating is enhanced.

These experimental results indicate that the catecholamine modification approach proposed in this study promotes the performance and stability of sol–gel coatings. The corrosion resistance of the CA/MPD-modified coating is significantly better than that of the unmodified sol–gel sample. The balance between coating thickness and compact stability plays a crucial role, and selecting an appropriate concentration of CA/MPD solution is necessary to optimize the modification effect.

### 2.2. Surface Characterization of CA/MPD@sol–gel

**Figure 4:**
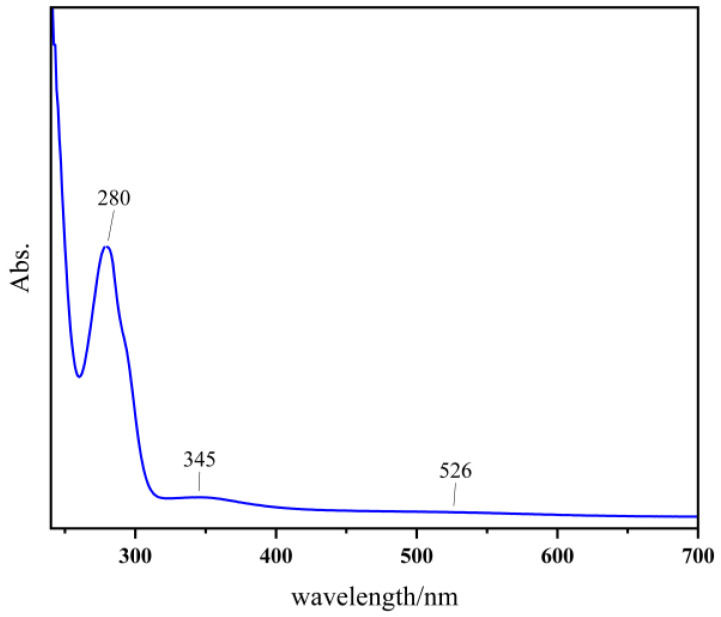
UV-Vis absorption spectra of CA/MPD@sol–gel coatings. The results confirmed that the sol–gel coating was successfully modified by CA/MPD.

The thickness, composition, and microstructure of organic anti-corrosion coatings are key factors determining their performance. To study the reaction mechanism of CA/MPD on the sol–gel surface, a small amount of modified CA/MPD solution was diluted 25 times and analyzed using ultraviolet-visible spectroscopy (UV-Vis) to investigate the polymerization process (Figure 4). The results showed an absorption peak at 280 nm, indicating the presence of quinone species, suggesting that the diphenol structure of catechol was oxidized to quinone. Additionally, the broad band at 345 nm represents the n-π* transition of the C=N double bond generated by the Schiff base reaction between CA and MPD. The increase in absorbance at 526 nm is associated with the Michael addition reaction between catechol and m-phenylenediamine.

**Figure 5:**
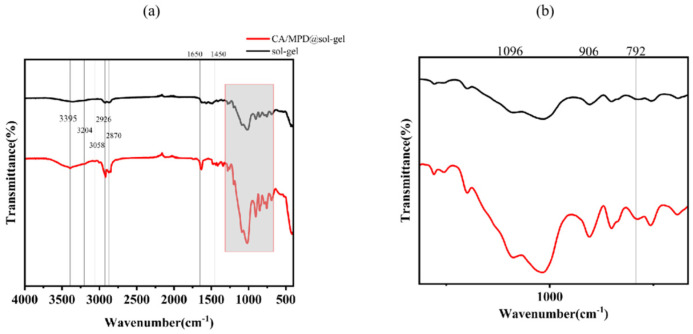
FT-IR absorption spectra of CA/MPD@sol–gel coatings. (**a**) Full-spectrum range (4000–400 cm^−1^); (**b**) enlarged local range. Infrared findings strongly suggest that CA/MPD, drawing inspiration from the chemistry of mussels, effectively altered the sol–gel coating. 792 cm^−1^: Symmetric stretching of Si-O-Si. 1096 cm⁻^1^: Symmetric stretching vibrations of Si-O-Si. 906 cm^−1^: Epoxy groups. 2870 cm^−1^ and 2926 cm^−1^: Symmetric and asymmetric stretching vibrations of C-H in GPTMS. 3395 cm^−1^: -OH groups introduced by catechol modification. 1650 cm⁻^1^ to 1450 cm⁻^1^: Characteristic bands of aromatic rings. 3204 cm^−1^ and 3058 cm^−1^: Catechol groups. 1150 cm^−1^ to 1200 cm^−1^: C-N groups.

Fourier transform infrared spectroscopy (FT-IR) analysis was performed on the CA/MPD@sol–gel coating to study whether the sol–gel was successfully modified by CA-MPD (Figure 5). The CA/MPD@sol–gel retains the characteristic infrared bands of the sol–gel, including symmetric stretching of Si-O-Si (792 cm^−1^), asymmetric stretching vibrations of Si-O-Si (1096 cm^−1^), and epoxy groups (906 cm^−1^) [30]. Furthermore, bands at 2870 cm^−1^ and 2926 cm^−1^ reflect the symmetric and asymmetric stretching vibrations of C-H in GPTMS, respectively. The peak at 3395 cm^−1^ of the CA/MPD@sol–gel reflects the -OH groups introduced by catechol modification. Additional characteristic bands of aromatic rings are observed at 1650 cm^−1^ to 1450 cm^−1^, and catechol groups at 3204 cm^−1^ and 3058 cm^−1^ [26]. Absorption bands of C-N groups are present from 1150 cm^−1^ to 1200 cm^−1^, potentially overlapping with the Si-O-Si characteristic bands, making them difficult to observe separately. These infrared results provide strong evidence that CA/MPD, inspired by mussel chemistry, successfully modified the sol–gel coating.

**Figure 6:**
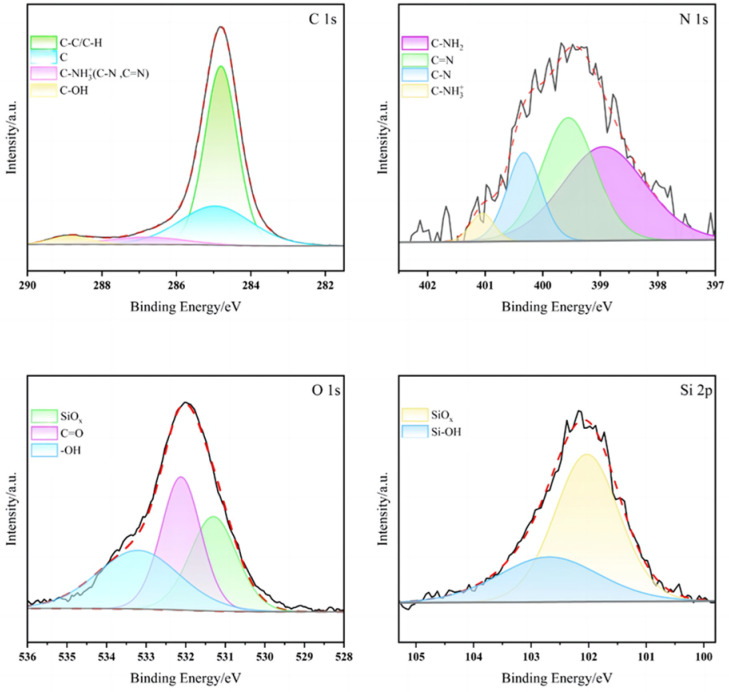
XPS spectra of CA/MPD@sol–gel sample showing C 1s, N 1s, O 1s, and Si 2p peaks. The characteristic Si-OH and SiO_x_ of the sol–gel were also detected on the surface, indicating that CA/MPD catecholamine substances did not merely form a new film on the sol–gel surface but interacted with it, altering the surface structure of the sol–gel coating.

X-ray photoelectron spectroscopy (XPS) tests were conducted on the CA/MPD@sol–gel coating to confirm the successful modification of the sol–gel by CA/MPD. The XPS spectra of C1s, N1s, O1s, and Si2p (Figure 6) show the fitted peaks corresponding to C-NH_2_, C-NH_3_⁺, C-N, and C=N bonds, which are absent in the unmodified sol–gel, providing strong evidence for the incorporation of amine groups in the CA/MPD@sol–gel sample. The characteristic Si-OH and SiOx of the sol–gel are also detected on the surface, indicating that CA/MPD catecholamine substances did not merely form a new film on the sol–gel surface but interacted with it, altering the surface structure of the sol–gel coating. The XPS results strongly confirm the successful modification of the sol–gel coating by CA/MPD.

**Figure 7:**
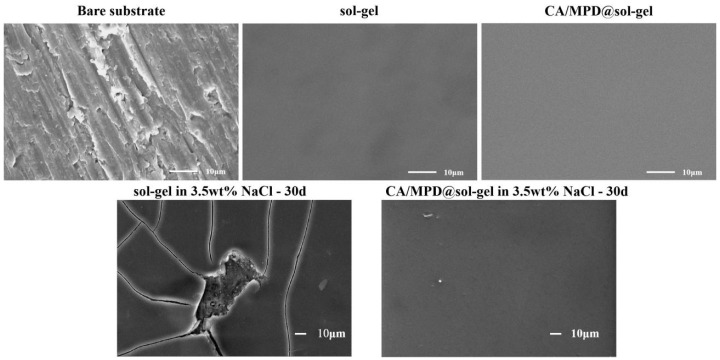
SEM images of surfaces of aluminum alloy substrate, sol–gel, and CA/MPD@sol–gel coatings, and the surface morphology after 30 days of immersion in 3.5 wt% NaCl solution No obvious defects were found in CA/MPD-modified sol–gel coating.

Figure 7 shows the scanning electron microscopy (SEM) results of the surfaces of the aluminum alloy substrate, sol–gel samples, and CA/MPD@sol–gel samples. The 3003 aluminum alloy surface polished with 1200-grit sandpaper appears relatively rough, with visible polishing marks. In contrast, the sol–gel and CA/MPD@sol–gel coatings provide a smoother surface that uniformly covers the polished aluminum alloy, without noticeable defects or cracks. After 30 days of immersion in a 3.5 wt% NaCl solution, the SEM results of the coating surface show significant cracking and defects in the sol–gel coating, which, combined with the EIS results, indicates a loss of corrosion protection ability. Meanwhile, the CA/MPD@sol–gel coating remains relatively smooth, with only a few localized damage points, maintaining good corrosion protection ability according to the EIS results.

**Figure 8:**
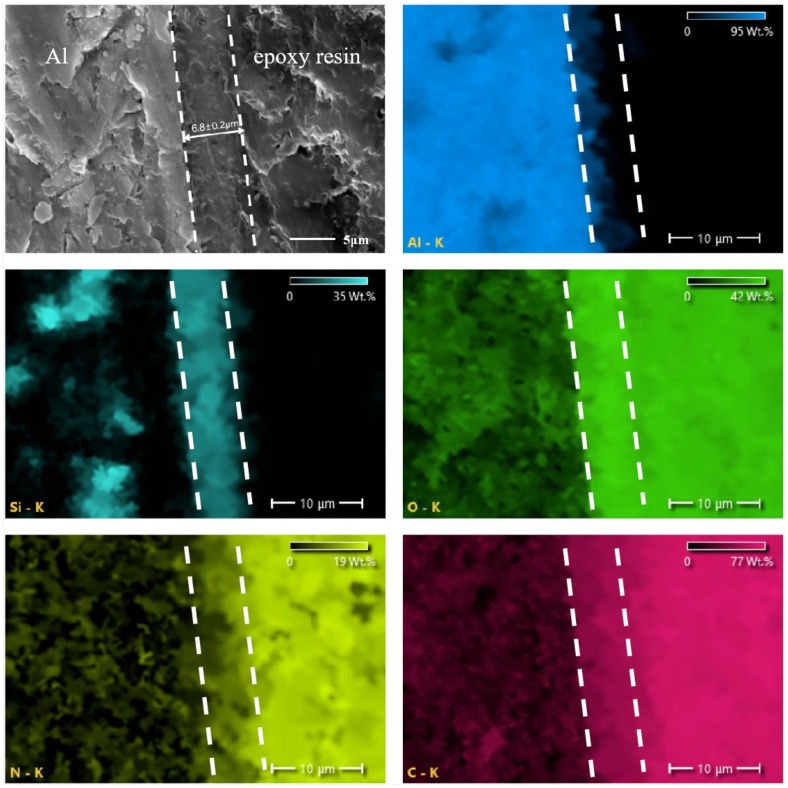
Cross-sectional SEM and EDS analysis results of CA/MPD@sol–gel coating. The results indicated a uniform distribution of nitrogen across the cross-section.

Figure 8 presents the SEM and EDS results for the cross-section of the CA/MPD@sol–gel coating. The SEM and EDS results indicate that the left side is the Al Alloy substrate, the middle is the CA/MPD@sol–gel coating with a thickness of approximately 6.8 μm, and the right side is the epoxy resin layer used to protect the coating during cross-sectional polishing. The high oxygen content detected on the right side is due to the epoxy resin. The EDS results show that nitrogen is evenly distributed throughout the cross-section, which, combined with the XPS results, confirms that CA/MPD diffused into the sol–gel coating’s micropores or nanopores, filling the coating defects.

### 2.3. Investigation of the Protective Mechanism of CA/MPD@sol–gel

Extensive studies have shown that sol–gel coatings struggle to withstand long-term corrosion tests [20,21,24,31,32]. The results of this study also confirm this pattern: unmodified sol–gel coatings are generally damaged over time in corrosive environments. This damage is likely due to the microporous or nanoporous microstructure within the coating, which allows corrosive media to diffuse through these pores and reach the interface between the sol–gel coating and the aluminum alloy substrate.

However, this study demonstrates that sol–gel coatings modified with CA/MPD do not exhibit signs of coating failure even after 30 days of corrosion testing, and their EIS values decline at a relatively slow rate over time. Combined with the characterization analysis of CA/MPD@sol–gel, it can be inferred that the nanoporous glass-like polymer grid of the sol–gel is filled with CA/MPD catecholamine materials. This indicates that CA/MPD is not merely attached to the sol–gel surface as a new film but diffuses into the sol–gel’s internal microporous or nanoporous structure, interacts with the sol–gel, and changes the coating structure. This increases the overall density of the coating, forming a denser protective layer, effectively inhibiting the diffusion of chloride ions and water molecules to the coating-substrate interface, thereby enhancing the coating’s corrosion protection performance.

**Figure 9:**
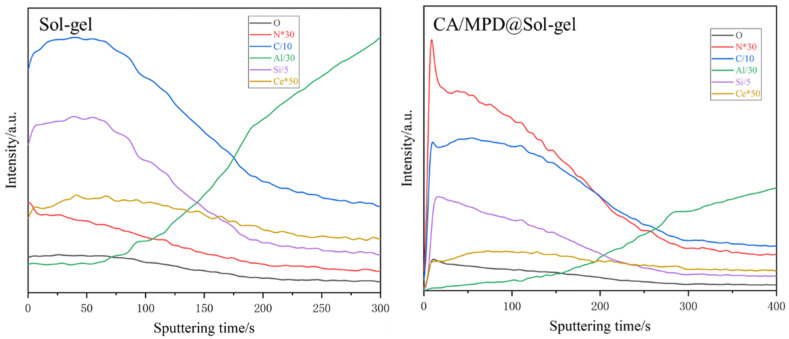
Depth profiling results of elemental distribution in the cross-section of CA/MPD@sol–gel coating by glow discharge optical emission spectroscopy (GD-OES). The relative amount of nitrogen in the cross-section of the modified coating showed a notable increase, suggesting that CA/MPD effectively penetrated the sol–gel coating during immersion and modified the entire coating.

**Figure 10:**
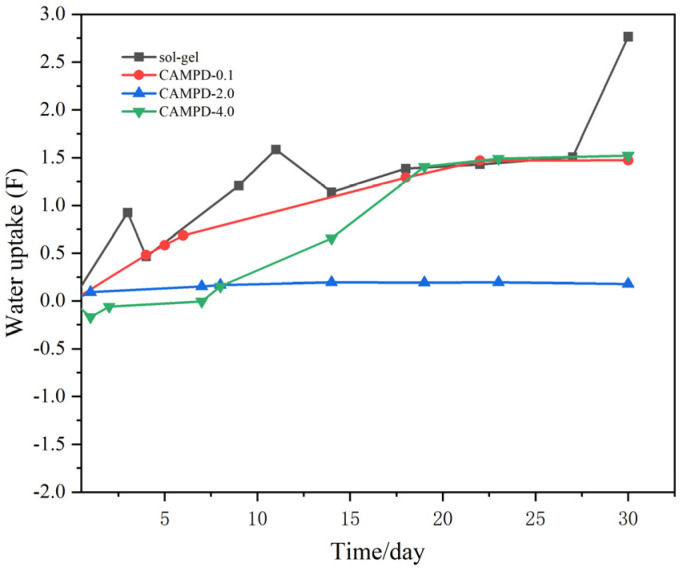
Comparison of water permeability rates for different coatings. This suggested that the penetration modification of CA/MPD greatly enhanced the compactness of the coating.

As shown in the experimental data in Figure 9, the relative proportion of nitrogen in the cross-section of the modified coating increased significantly, indicating that CA/MPD had successfully penetrated the sol–gel coating during immersion and modified the entire coating. Furthermore, as demonstrated in Figure 10, the modified CA/MPD-2.0 coating exhibited a significantly lower water permeability rate during prolonged immersion. This suggests that the penetration modification of CA/MPD can greatly enhance the compactness of the coating.

Additionally, this study explored the effect of CA/MPD concentration on the catecholamine-modified sol–gel. At low concentrations, the sol–gel coating’s corrosion resistance and stability showed only a slight improvement, likely due to the insufficient amount of CA/MPD to uniformly and comprehensively fill the microstructural defects in the sol–gel coating. As the concentration of CA-MPD increases, the electrochemical impedance of the coating shows a significant improvement, with good coating stability. However, at excessively high concentrations, the degree of enhancement in electrochemical impedance is reduced. This could be due to the high concentration of CA/MPD solution adversely affecting the base thickness of the sol–gel coating. Excessive diffusion of catecholamine material into the internal microporous or nanoporous structure increases internal stress, causing new defects that weaken the coating’s protective ability.

This study proposes a catechol-m-phenylenediamine-modified sol–gel method, which shows feasibility and practical significance for potential applications in industrial production. However, this study used only a 3.5 wt% NaCl solution to simulate a marine corrosion environment, which may not fully represent the actual service conditions of aluminum alloys. Therefore, the performance of coatings in different environmental media still needs to be discussed. Additionally, this experiment only set three concentrations of CA/MPD solutions (0.1 mg/mL, 2 mg/mL, 4 mg/mL) and found that the modification effect initially increases and then decreases with concentration. Future applications should consider more concentration gradients under specific corrosion environments to understand their impact on modification effects.

## 3. Materials and Methods

### 3.1. Materials and Sample Preparation

The substrate material used in this study was a commercial 3003 aluminum alloy plate, with its chemical composition shown in Table 1. The surface of the aluminum alloy samples was polished using silicon carbide sandpaper (from 600 to 1200-grit), then cleaned with ethanol, ultrasonicated in anhydrous ethanol for 300 s, and subsequently dried in an oven at 60 °C.

The silica sol used in this experiment was prepared by mixing deionized water, tetraethyl orthosilicate (TEOS, MERYER > 99%), 3-(glycidyloxypropyl)trimethoxysilane (GPTMS, Macklin 98%), and ethanol in a volumetric ratio of 1:1:3:5. The mixture was stirred at 25 °C and 500 rpm for 30 min. Afterward, cerium nitrate was added to the solution to a concentration of 0.01 M, and the stirring continued for another 20 min. Then, 2 drops of 0.3 M acetic acid solution were added, and the mixture was stirred for an additional 60 min before being left to stand in the air for 14 h. The sol–gel solution prepared by the above steps was applied to the surface of the pre-polished aluminum alloy material. The coated samples were left undisturbed for 30 min to ensure uniform distribution, then placed in an oven and cured at 100 °C for 180 min. The samples were then allowed to cool naturally to room temperature. These samples were designated as sol–gel samples.

For modification of the sol–gel samples, catechol (Catechol, 99.5%, denoted as CA) and m-phenylenediamine (m-Phenylenediamine, 99%, denoted as MPD) were used to synthesize catecholamine substances, as illustrated in Figure 3. Initially, CA and MPD solids were weighed at a 1:1 ratio and dissolved in deionized water. The pH of the solution was adjusted to 8.5, and tris(hydroxymethyl)aminomethane (Tris, AR, denoted as Tris) was added to a concentration of 1.2144 mg/mL. The sol–gel samples were then prepared by sealing all areas except the surface requiring modification with electrical insulating tape and immersing them in the Tris buffer solution containing CA/MPD. The samples were stirred with a magnetic stirrer at 500 rpm at room temperature for 14 h. They were then cleaned using ultrasonic treatment in anhydrous ethanol and dried. To study the effect of CA/MPD dosage on the modification of sol–gel coatings, three different concentrations of CA/MPD solutions (0.1 mg/mL, 2 mg/mL, and 4 mg/mL) were prepared for modification, and the modified samples were designated as CA/MPD-0.1@sol–gel, CA/MPD-2@sol–gel, and CA/MPD-4@sol–gel, respectively.

### 3.2. Corrosion Testing and Sample Characterization

Corrosion tests were conducted using a traditional three-electrode vertical cell system. The samples were placed at the bottom of an electrochemical cell containing 3.5 wt% NaCl solution, with a coated surface area of 1 cm^2^ exposed to the solution. The back side of the samples was connected with conductive copper adhesive to form the working electrode, with a platinum sheet as the counter electrode and a saturated calomel electrode (SCE) as the reference electrode. Before conducting electrochemical impedance spectroscopy (EIS, Corrtest 310H, Wuhan, China), the open circuit potential (OCP) was maintained for 30 min to stabilize the interface between the sample and the solution. EIS and potentiodynamic polarization tests were also conducted on the blank aluminum alloy substrate (after polishing). The polarization test was conducted from −0.5 V to 1 V versus OCP, with a scan rate of 1 mV/s. The EIS test parameters included an AC voltage of 10 mV rms, frequency range from 1 MHz to 10 mHz, with 7 points per decade. A polarization testing was also performed on sol–gel samples under the same conditions as the blank aluminum alloy. Four groups of samples—sol–gel, CA/MPD-0.1@sol–gel, CA/MPD-2@sol–gel, and CA/MPD-4@sol–gel—were subjected to long-term EIS testing using the same parameters as for the blank aluminum alloy.

Fourier transform infrared spectroscopy (FT-IR, PerkinElmer, Waltham, MA, USA) was used to analyze the functional groups in the sample coatings, with a scanning range of 4000 cm^−1^ to 400 cm^−1^. Ultraviolet-visible spectrophotometry (UV-Vis, Shimadzu, Japan) was employed to study the types of chemical bonds formed, with a scanning wavelength range set from 200 nm to 700 nm. Scanning electron microscopy (SEM, Thermo Fisher Scientific, Waltham, MA, USA) was used to observe the surface morphology and cross-sectional features of the aluminum alloy substrate and coatings, complemented by energy dispersive X-ray spectroscopy (EDS, Oxford instruments, High Wycombe, UK) for elemental analysis. Due to the poor conductivity of the coatings, a gold sputtering process was applied for 30 seconds before SEM and EDS testing. X-ray photoelectron spectroscopy (XPS, Thermo Fisher Scientific, Waltham, MA, USA) was used to determine the chemical states of the elements on the sample surfaces.

## 4. Conclusions

This study designed and prepared an innovative, green CA/MPD-modified sol–gel coating with a thickness of approximately 6.8 µm. The corrosion protection performance and stability of the CA/MPD@sol–gel coating were significantly improved compared to the unmodified sol–gel coating. The conclusions are as follows:

1. Long-term EIS tests on the modified coating showed that after 30 days of testing, the catecholamine-modified sol–gel coating still maintained good protective performance. This result indicates that the CA/MPD@sol–gel-modified coating significantly improves the coating’s stability and low-frequency impedance in EIS tests, enhancing the sol–gel coating’s protective performance.
2. The modification effect of CA/MPD on the sol–gel changes with increasing concentration, initially rising and then falling. Very low or very high concentrations of CA/MPD are unfavorable for the coating’s protective performance. Therefore, an appropriate concentration of CA/MPD should be selected for modifying the coating.
3. The CA-MPD@sol–gel material was characterized using ultraviolet-visible absorption spectroscopy (UV-Vis), Fourier transform infrared absorption spectroscopy (FT-IR), scanning electron microscopy (SEM), energy-dispersive X-ray spectroscopy (EDS), and X-ray photoelectron spectroscopy (XPS). The CA/MPD adsorbed on the sol–gel surface and diffused into the sol–gel coating, forming chemical bonds. By filling the microporous or nanoporous structure inside the sol–gel, the modification filled the microstructural defects of the coating, enhancing its compactness and effectively inhibiting the diffusion of solvent molecules and corrosive particles, such as chloride ions, to the aluminum alloy surface, thereby enhancing the corrosion protection performance of the sol–gel coating.

## Figures and Tables

**Table 1:** Chemical composition of 3003 aluminum alloy.

Element	Mn	Cu	Fe	Si	Zn	Al
wt%	1.0–1.5	0.05–0.2	0.7	0.6	0.1	Bal.

## Data Availability

The data presented in this study are available on request from the corresponding author.
